# Effective Modulating Brassinosteroids Signal to Study Their Specific Regulation of Reproductive Development and Enhance Yield

**DOI:** 10.3389/fpls.2019.00980

**Published:** 2019-07-26

**Authors:** Song-Hao Zu, Yu-Tong Jiang, Li-Qin Hu, Yan-Jie Zhang, Jin-Hui Chang, Hong-Wei Xue, Wen-Hui Lin

**Affiliations:** ^1^School of Life Sciences and Biotechnology, The Joint International Research Laboratory of Metabolic and Developmental Sciences, Shanghai Jiao Tong University, Shanghai, China; ^2^School of Agriculture and Biology, Shanghai Jiao Tong University, Shanghai, China; ^3^National Key Laboratory of Plant Molecular Genetics, CAS Center for Excellence in Molecular Plant Sciences, Institute of Plant Physiology and Ecology, Chinese Academy of Sciences, Shanghai, China

**Keywords:** brassinosteroid, architecture, organ size, planting density, yield

## Abstract

Brassinosteroid (BR) is a family of bioactive steroid hormones that plays vital roles in plant growth and development. The BR-mediated regulation of plant growth and architecture has been well studied. However, relatively few studies have investigated the BR-related regulation of reproductive development because of the difficulties in excluding non-specific regulation and secondary responses from severe vegetative phenotypes and poor nutritional status. Furthermore, differentially regulating the BR signal in vegetative and reproductive organs is problematic. Thus, establishing a method for modulating the BR signal only in reproductive organs or during reproductive developmental stages will be beneficial. Additionally, the utility of BR applications for crop production is limited because of deleterious side-effects, including the associated decrease in the planting density and lodging resistance. Moreover, enhancing the BR signal may lead to feedback inhibition. In this study, we developed a transformation system for modulating the BR signal differentially during reproductive and vegetative developmental stages. This system involves transformations with different combinations of a reproductive tissue-specific promoter, coding sequences that increase or decrease the BR signal, and various genotypic backgrounds with enhanced or decreased BR signals. The enhanced BR signal generated in transformants was targeted to reproductive organs without affecting vegetative organs. This system may be useful for studying the BR-specific regulation of plant reproductive development and shows promise for optimizing seed yield.

## Introduction

Phytohormones play essential roles in the regulation of seed development, seed size, seed weight, and crop yield. Brassinosteroid (BR) is plant steroid hormones that affect cell elongation and division, tissue differentiation, organogenesis, reproductive development, photomorphogenesis, and immunity (Morinaka et al., 2006; Sakamoto et al., 2006). In rice, BR can also decrease the amount of residues of common organophosphorus, organochlorine, and carbamate pesticides (Zhou et al., 2015) and induce crop tolerance to these pesticides (Ahammed et al., 2012a,b).

The BR signal transduction pathway has been well studied. The BR receptor BRASSINOSTEROID INSENSITIVE1 (BRI1, a cell-surface receptor kinase) (Li et al., 2001a) and the BR-induced transcription factors BRASSINAZOLE RESISTANT1 (BZR1) (Wang et al., 2002) and BRI1-EMS-SUPPRESSOR1 (BES1) (Yin et al., 2002) are key components for activating the BR signal. BRASSINOSTEROID INSENSITIVE2 (BIN2), a GSK3-like kinase, is the main repressor of the BR signal (Li et al., 2001b; He et al., 2002). The strong alleles of *BRI1*, such as *bri1-116*, show severe phenotypes of strongly dwarf, very small and round leaves, and almost complete sterility (Li and Chory, 1997). In contrast, the weak alleles of *BRI1*, such as *bri1-5*, exhibit semi-dwarfism, with small and round leaves as well as decreased fertility (Li et al., 2001a). A gain-of-function mutant of *BIN2*, *bin2-1*, that exhibits phenotypes associated with decreased BR signal levels, similar to the *bri1* mutant (Li et al., 2001b). A gain-of-function mutant of *BZR1*, *bzr1-1D*, which phenotype is associated with enhanced BR signaling (Wang et al., 2002; He et al., 2005). Compared with wild-type plants, this mutant produces longer and curved petioles, longer and kinked inflorescence stems, larger floral organs, and more seeds. The increased BZR1 dephosphorylation in the *bzr1-1D* mutant is indicative of high BZR1 activity and higher BR signals.

Brassinosteroid positively regulates seed development and seed size/weight by modulating the transcription of *SHORT HYPOCOTYL UNDER BLUE 1* (*SHB1*)–*MINISEED 3* (*MINI3*)–*HAIKU2* (*IKU2*), *APETALA 2* (*AP2*), and *AUXIN RESPONSE FACTOR 2* (*ARF2*) (Schruff et al., 2006; Ohto et al., 2009; Zhou et al., 2009; Jiang et al., 2013). Additionally, BR positively regulates ovule development and seed production (Huang et al., 2013), implying BR enhances seed yield. Brassinosteroid is well known to regulate rice architecture and grain yield (Zhang et al., 2018), and are important for regulating rice plant height (Fujioka and Yokota, 2003). The *D11* gene encoding a cytochrome P450 (CYP724B1), functions in BR biosynthesis and affects rice plant height (Tanabe et al., 2005). The BR-deficient mutant *d2* has a smaller leaf angle (Hong et al., 2003), implying that BR has a significant impact on leaf bending. In another BR-deficient mutant, *brd1*, the internodes essentially do not elongate (Hong et al., 2002). Increased BR contents can lead to the production of more tillers, larger spikes, and more grains per spike (Wu et al., 2008). Brassinosteroid also positively regulates rice seed/grain size/weight (Hong et al., 2005; Tanabe et al., 2005; Morinaka et al., 2006; Sahni et al., 2016). Although a number of specific regulators and different regulatory mechanisms have been identified in rice, including *ENHANCED LEAF INCLINATION AND TILLER NUMBER1* (*ELT1*, Yang et al., 2017), *RICE LEAF AND ILLER ANGLE INCREASED CONTROLLE*R1 (OsLIC1, Wang et al., 2008), and *DWARF AND TILLERING* (DLT, Tong et al., 2009), BR signal transduction and BR-regulated growth and development are essentially conserved in Arabidopsis, rice, soybean, and other plant species (Nakamura et al., 2006; Sakamoto et al., 2006; Bai et al., 2007; Tanaka et al., 2009; Zhang et al., 2016). Arabidopsis BR-related mutants are useful for identifying the BR signal regulators in other species.

Brassinosteroid also regulates the development of pollen grains (Ye et al., 2010), ovules (Huang et al., 2013), the embryo sac (Pérez-España et al., 2011), and seeds (Jiang et al., 2013). However, investigating the BR-specific regulation of plant reproductive development is difficult because severe vegetative phenotypes and poor nutrient accumulation in strong alleles of BR-deficient or insensitive mutants would affect reproductive development indirectly. Therefore, it is difficult to exclude the possibility of a secondary response or non-specific regulation. Establishing a transformation system that enables the differential modulation of the BR signal in various organs or developmental stages may be much helpful. Such a system may be relevant for increasing crop production, especially rice.

Increasing the total yield is an important aim of crop breeding. The total yield depends on the yield of individual plants and the planting density. The grain yield of individual rice plants may be improved by enhancing the BR signal, but the growing area of an individual plant would also increase, thereby leading to decreased planting density. Furthermore, plant height would increase and lodging resistance would decrease, which would negatively affect grain yield. Previous studies revealed that enhancing the BR signal contributes to improved nutritional status, greater efficiency of carbohydrate transport from the source to the sink, and increased grain yield. However, an overall increase in BR content also results in larger laminar joints and increased height, leading to decreased lodging resistance and planting density (Choe et al., 2001; Wu et al., 2008). These negative side-effects may limit the utility of BR applications for agricultural production. A system that specifically modulates BR signals in reproductive organs may be highly beneficial for increasing the total rice yield. In this study, we established a transformation system for modulating the BR signal during reproductive development. This system could be used to investigate the BR-specific regulation of plant reproductive development and optimize seed yield.

## Materials and Methods

### Plant Materials, Growth Conditions, and Transformation Procedure

The *pSTK*::*GUS* (pBI101.3) vector was transformed into wild-type Arabidopsis plants (Columbia ecotype; Col) to examine *STK* promoter activity. The *pSTK*::*bzr1-1D*-*GUS* (pBI101.3) and *pSTK*::*bzr1-1D*-*GFP* (pCAMBIA1302) vectors were transformed into *bri1-5* plants (Wassilewskija ecotype) to enhance the BR signal in reproductive organs. Similarly, the *pSTK*::*bzr1-1D*-*GFP* vector was transformed into *bin2-1* ± plants (Col ecotype) to enhance the BR signal in reproductive organs. The *pSTK*::*bin2-1*-*GUS* vector was transformed into *bzr1-1D* and *DWF4-OX* (Col ecotype) plants to repress the BR signal in reproductive organs. Additionally, the *pSTK*::*bin2-1*-*GFP* vector was transformed into *bzr1-1D*, *DWF4-OX*, and wild-type (Col ecotype) plants to repress the BR signal in reproductive organs.

All plants were grown in a greenhouse maintained at 22°C under a 16-h light/8-h dark photoperiod. *Agrobacterium*-mediated transformation was performed using the floral dip method (Clough and Bent, 1998). The positive clones were cultured for 10 h at 28°C in YEP medium (10 g/L peptone, 10 g/L yeast extract, and 5 g/L NaCl, pH 7.2) supplemented with 50 mg/L rifampicin and 50 mg/L kanamycin. The *Agrobacterium* cells were collected and diluted with 5% sucrose solution containing 0.02% (v/v) Silwet L-77 (SL77080596, GE) to OD_600_ 0.8–1.0. The Arabidopsis inflorescence apices were dipped in the mixture buffer, sealed, and kept in the dark overnight.

### Construction of Vectors

The 2,008 bp promoter and 1,085 bp coding sequence (gDNA) of *STK* was amplified by PCR with KOD-FX DNA polymerase and primers STK-F and STK-R, and cloned into the pBI101.3 vector (digested with *Bam*HI) to generate the *pSTK*::*GUS* vector.

To obtain the *bzr1-1D* coding sequence, the former 1,055 bp and the latter 321 bp gDNA of BZR1 were amplified with KOD-FX DNA polymerase. The specific primer pairs containing a point mutation were BZR1-F and bzr1-1D-R, and bzr1-1D-F and BZR1-R, respectively. We then amplified 1,355 bp gDNA of *bzr1-1D* with the specific primers BZR1-F and BZR1-R by fusion PCR.

To insert *pSTK*::*bzr1-1D* in the pBI101.3 vector, *pSTK* was amplified with the primer pair STK-F and STK-R1, and *bzr1-1D* was amplified with the primer pair bzr1-1D-F1 and bzr1-1D-R1. *STK-bzr1-1D* was amplified with the primer pair STK-F and bzr1-1D-R1 by fusion PCR, after which the amplicon was cloned into pBI101.3 (digested with *Bam*HI) to generate the *pSTK*::*bzr1-1D*-*GUS* vector.

To generate the *pSTK*::*bzr1-1D*-*GFP* construct, STK-*bzr1-1D* was amplified with the primer pair STK-F1 and bzr1-1D-R2 using the *pSTK*::*bzr1-1D*-*GUS* vector as the template, and then was cloned into pCAMBIA1302 (digested with *Eco*RI and *Noc*I) to generate the *pSTK*::*bzr1-1D*-*GFP* vector.

Similar to *bzr1-1D*, we amplified *bin2-1* sequence by fusion PCR, and then constructed the pCAMBIA1302 *pSTK*::*bin2-1*-*GFP* and pBI101.3 *pSTK*::*bin2-1*-*GUS* vectors. All primer sequences are shown in Supplementary Table S1.

### qRT-PCR Assay

The quantitative real-time PCR (qRT-PCR) procedure was carried out as described previously (Zhang et al., 2016). Total RNA was extracted from 15-day-old rosette leaves and the apex of 40-day-old inflorescences. *CPD* sequence was amplified with CPD RT-F and CPD RT-R. *DWF4* sequence was amplified with DWF4 RT-F and DWF4 RT-R. *bzr1-1D-GFP* sequence was amplified with bzr1-1D RT-F and GFP RT-R. The *ACTIN* gene was amplified as an internal reference using the primers ACTIN RT-F and ACTIN RT-R. The procedure was 40 cycles of 94°C for 10 s, 60°C for 15 s, and 72°C for 20 s. Primer sequences are shown in Supplementary Table S2. The experiments were biologically repeated for 3 times and data are shown as means ± SD (*n* = 3).

### β-Glucuronidase (GUS) Staining Assay

The inflorescence apex of *pSTK::GUS* lines was dipped in GUS staining solution and vacuum-infiltrated for 30 min, maintained in the GUS staining solution at 37°C overnight, then chlorophyll was eluted with 75% alcohol twice. The stained tissues were observed with a Leica S8APO stereomicroscope and photographed with a Leica DFC450 digital camera.

### Identification and Characterization of Transgenic Lines

The *pSTK*::*GUS*, *pSTK*::*bzr1-1D*-*GUS*, and *pSTK*::*bin2-1*-*GUS* transgenic lines were verified with primers 77-R and GUS. The *pSTK*::*bzr1-1D*-*GFP* and *pSTK*::*bin2-1*-*GFP* transgenic lines were verified with the primer pairs 77-R and GFP. Primer sequences are shown in Supplementary Table S3.

The phenotype of 21-day-old plants and the leaf shape of transgenic lines were photographed using a Canon EOS60D digital camera. The siliques from different lines were observed with a Leica S8APO stereomicroscope and photographed with a Leica DFC450 digital camera and the experiments were biologically repeated for 3 times and data are shown as means ± SD (*n* = 15). The seed yield (seed weight per plant) was measured by an analytical balance and the experiments were biologically repeated for 3 times and data are shown as means ± SD (*n* = 5).

## Results

### Designing Transgenic Plants to Modulate the BR Signal in Reproductive Organs

**FIGURE 1:**
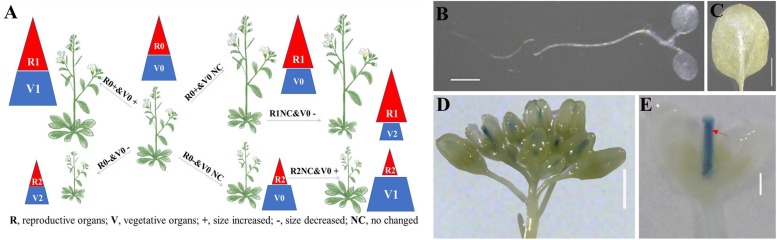
Design of transformation system for differential manipulation of brassinosteroid (BR) signal in reproductive and vegetative organs. **(A)** 0: Original BR signal and normal organ size; 1: increased BR signal and organ size; 2: decreased BR signal and organ size, R: reproductive organs, V: vegetative organs, +: size increased, -: size increased, NC: no changed **(B–E)** β-Glucuronidase (GUS) staining assay of *pSTK*::*GUS* (pBI101.3) transgenic lines. The GUS signal was detected in the cotyledons **(B)**, rosette leaves **(C)**, inflorescence apex **(D)**, and pistil and ovules **(E)**, bar = 1 cm. Red arrow indicates ovules.

The normal BR signal positively regulates plant growth and development in both vegetative and reproductive organs (Figure 1A, R1V1 and R0V0). The reproductive development of plants with the strong alleles of BR-deficient/insensitive mutants was severely impacted. However, we could not exclude the possibility that the phenotypes were the result of the non-specific regulation of poor vegetative growth and poor nutrient accumulation. To investigate the BR-specific regulation of plant reproductive development, we generated transgenic plants in which the BR signal was specifically modulated in the reproductive organs during the reproductive developmental stages (Figure 1A, R1V0 and R2V0). To examine the relevant regulatory processes and to distinguish BR signals in various organs, we also generated transgenic lines with the opposite modification to the BR signal (Figure 1A, R1V2 and R2V1).

The transformation system developed in this study may be useful for improving crop production. Specifically, R1V0 enhanced reproductive development without influencing vegetative growth, thereby avoiding side-effects. Additionally, R1V2 was the optimal combination, which resulted in increased reproductive organ size and appropriately decreased vegetative organ size. Developing the transformation system involved the following three steps. The first step was identifying specific promoters that are active in reproductive tissues. The second step was constructing the coding sequences for the efficient modulation of the BR signal. The final step was selecting plants in which the BR signal was modulated for transformation.

### Selection of Tissue-Specific Promoters

Selecting tissue-specific promoters that are sufficiently active is an important step. Previous studies indicated that MADS-box genes, such as *SEPALLATA 1* (*SEP1*), *SEP2*, *SEP3*, *AGAMOUS* (*AG*), *SHATTERPROOF 1* (*SHP1*), *SHP2*, and *SEEDSTICK* (*STK*), are expressed in reproductive organs and control flower and ovule identity (Bowman et al., 1989; Liljegren et al., 2000; Pelaz et al., 2000; Favaro et al., 2003; Pinyopich et al., 2003). For example, *STK* was predicted to be highly transcribed (Supplementary Figure S1), mainly in the pistil and ovule. The results of GUS staining assays (Pinyopich et al., 2003; Kooiker et al., 2005; Nain et al., 2008) revealed that *STK* is only expressed in the septum and ovules. Consequently, we cloned the longest *STK* promoter into the pBI101.3 vector with a portion of genomic DNA (from -2,008 bp to 1,085 bp) to ensure accurate expression. We introduced the *pSTK*::*GUS* vector (pBI101.3) into wild-type Arabidopsis (Col) plants and assessed the transcription activity in a GUS staining assay. The results illustrated that the *STK* promoter was active in the septum and ovules, but not in the cotyledons and rosette leaves (Figures 1B–E), which was consistent with previous findings (*pSTK*::*GUS* in the pCAMBIA1300-H vector; Kooiker et al., 2005) (Supplementary Figure S2). Thus, the *STK* promoter would be useful for enhancing the BR signal in the septum and ovule. Additionally, the *STK* promoter-controlled vector appears to be a suitable tool for studying the BR-specific regulation of septum and ovule development.

### Identification of Appropriate Coding Sequences to Directly Regulate the BR Signal

We selected specific coding sequences to efficiently modulate BR signaling and responses. The pBI101.3 and modified pCAMBIA1302 (35S promoter removed) vectors were used to harbor the coding sequences driven by the *STK* promoter. The activity of the BR-induced transcription factor BZR1 directly influences BR signaling and responses, and consequently seed production (Huang et al., 2013; Jiang et al., 2013; Zhang et al., 2016). Thus, we constructed a coding sequence (P234L) to mimic the gain-of-function mutant of BZR1, *bzr1-1D*. Finally, we inserted the *pSTK*::*bzr1-1D* construct (Supplementary Figure S3) into the pBI101.3 (*pSTK*::*bzr1-1D*-*GUS*) and pCAMBIA1302 (*pSTK*::*bzr-1D*-*GFP*) vectors.

Using a similar method, we inserted the *pSTK*::*bin2-1* construct (Supplementary Figure S3) into the pBI101.3 (*pSTK*::*bin2-1*-*GUS*) and pCAMBIA1302 (*pSTK*::*bin2-1*-*GFP*) vectors to mimic the *bin2-1* mutant and decrease the BR signal in reproductive organs.

### Selection of Plants in Which the BR Signal Is Modulated for Transformation

We introduced the prepared vectors into wild-type Arabidopsis (Col) plants and observed the specifically enhanced or decreased BR signal in the reproductive organs of the transgenic lines. To investigate whether the BR signal in vegetative organs influences the BR-mediated regulation of reproductive development, we also transformed BR-insensitive mutants (*bri1-5* and *bin2-1*) (Li et al., 2001a,b), a BR-signal-enhanced line (*bzr1-1D*), and the transgenic line *DWF4-OX*, which overexpresses the BR biosynthesis gene *DWF4* (Choe et al., 2001).

### Specific Enhancement of the BR Signal in Reproductive Organs

**FIGURE 2:**
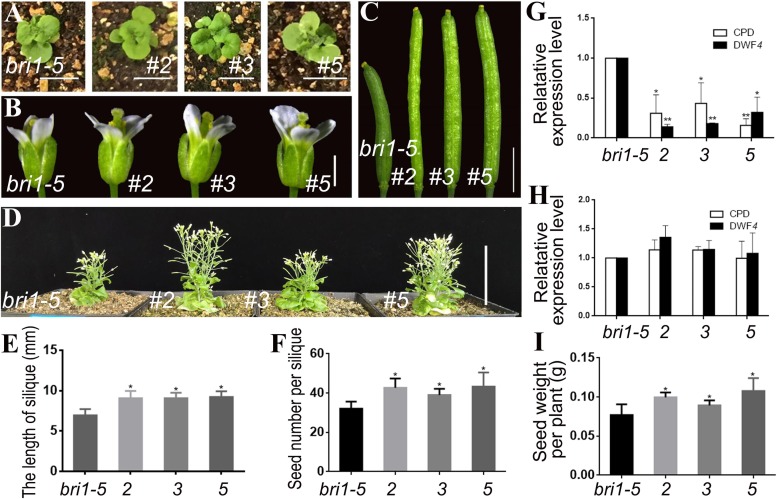
Phenotypic analysis of *pSTK*::*bzr1-1D*-*GUS* lines in *bri1-5* background. **(A)** Shape and size of rosette leaves, bar = 1 cm. **(B)** Floral organs, bar = 2 mm. **(C)** Siliques, bar = 2 mm. **(D)** Adult plants, bar = 5 cm. **(E,F)** Silique length and seed number. Bars represent the means ± SD of three biological replicates (*n* = 15). **(G,H)** Relative expression level of *CPD* and *DWF4* in inflorescence apex and rosette leaves. Values correspond to the arithmetic means ± SD of three biological replicates (*n* = 3). **(I)** Seed weight per plant. Bars represent the means ± SD of three biological replicates (*n* = 5). Asterisks represent significant differences (Student’s *t*-test, ^*^P < 0.05, ^∗∗^*P* < 0.01).

We transformed *pSTK*::*bzr1-1D*-*GUS* and *pSTK*::*bzr1-1D*-*GFP* into the *bri1-5* mutant plants to increase the BR signal in reproductive organs. Given that *bri1-5* is a weak allele that leads to smaller vegetative organs and not bad nutrient accumulation compared with wild-type plants, these transformations were expected to be appropriate for investigating whether the BR signal separately regulates vegetative and reproductive development. Lines 2, 3, and 5 were transgenic lines harboring *pSTK*::*bzr1-1D*-*GUS* (Figure 2). The shape and size of the rosette leaves in the transgenic lines were not significantly different from those of the *bri1-5* mutant (Figure 2A), indicating that *pSTK*::*bzr1-1D* did not distinctly modify vegetative growth. As expected, the floral organs in the transgenic plants were visibly larger than those of the *bri1-5* mutant (Figure 2B) and the siliques of the transgenic plants were 30% longer than those of the *bri1-5* mutant (Figures 2C,E). Lines 2, 3, and 5 had an average of 42.7, 39, and 43.3 seeds per silique, respectively, whereas the *bri1-5* mutant had 32 seeds per silique (Figure 2F). The seed weight per plant was higher for lines 2, 3, and 5 than for the *bri1-5* mutant (Figure 2I). The qRT-PCR results revealed that *CPD* and *DWF4* transcription levels decreased in reproductive organs, implying that the BR signal was enhanced in reproductive organs (Figure 2G). Moreover, there were no significant changes to the transcription of *CPD* and *DWF4* in vegetative organs (Figure 2H), suggesting that the transformation did not increase the BR signal in vegetative organs (i.e., the BR signal was regulated separately in the reproductive and vegetative organs).

**FIGURE 3:**
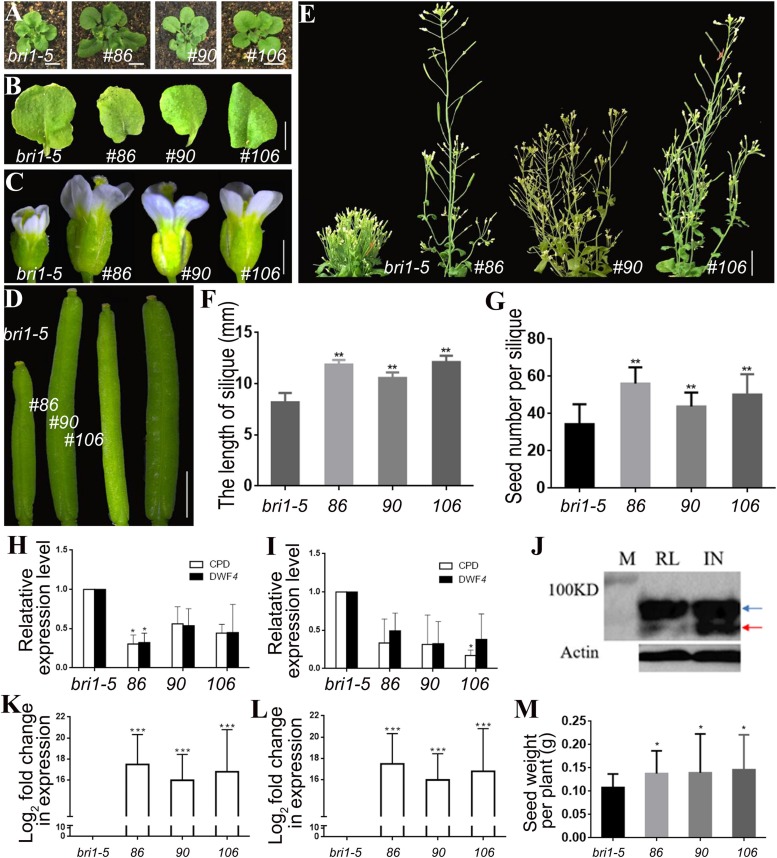
Phenotypic analysis of *pSTK*::*bzr1-1D*-*GFP* lines in *bri1-5* background. **(A,B)** Shape and size of rosette leaves, bar = 1 cm. **(C)** Floral organs, bar = 2 mm. **(D)** Siliques, bar = 2 mm. **(E)** Adult plants, bar = 2 cm. **(F,G)** Silique length and seed number. Bars represent the means ± SD of three biological replicates (*n* = 15). **(H,I)** Relative expression level of *CPD* and *DWF4* in inflorescence apex and rosette leaves. Values correspond to the arithmetic means ± SD of three biological replicates (*n* = 3). **(J)** Western-blot showing BZR1 (red arrow) and pBZR1 (blue arrow) in inflorescences (IN) and rosette leaves (RL). The protein marker (M) bands are 100 kD. Actin was the loading control. **(K,L)** Relative expression level of bzr1-1D-GFP in inflorescence apex and rosette leaves. Values correspond to the arithmetic means ± SD of three biological replicates (*n* = 3). **(M)** Seed weight per plant. Bars represent the means ± SD of three biological replicates (*n* = 5). Asterisks represent significant differences (Student’s *t*-test, ^*^P < 0.05, ^∗∗^P < 0.01, ^∗∗∗^P < 0.001).

Transgenic lines 86, 90, and 106 harboring *pSTK*::*bzr1-1D*-*GFP* were generated to assess the phosphorylation of the BZR1 protein and to verify the observed phenotypes. A western blot analysis involving GFP antibodies revealed more dephosphorylated BZR1 in the reproductive organs than in the vegetative organs of the transgenic lines, which indicated that BR was more active in reproductive organs than in vegetative organs (Figure 3J). Similar to lines 2, 3, and 5, the shape and size of the rosette leaves in lines 86, 90, and 106 were not significantly different from those of the *bri1-5* mutant (Figures 3A,B). The transgenic lines produced enlarged flowers and siliques (Figures 3C,D,F) and more seeds than the *bri1-5* mutant (Figure 3G). Additionally, lines 86, 90, and 106 were considerably taller than lines 2, 3, and 5 (i.e., longer inflorescence stems) (Figures 2D, 3E), which suggested that the enhanced BR signal spread from the expected organs to the inflorescence stem. The qRT-PCR results also revealed that *CPD* and *DWF4* transcription levels decreased in the vegetative and reproductive organs of the *pSTK*::*bzr1-1D*-*GFP* lines (Figures 3H,I), indicating that the BR signal was enhanced in vegetative and reproductive organs. These observations were in contrast to the response of *pSTK*::*bzr1-1D*-*GUS* transgenic lines, suggesting the more specific modulation of BR signal by pBI101.3 vector. Accompanied by the varied plant height, silique size and seeds number, the qRT-PCR data illustrated that the varied transcription of the introduced *bzr1-1D*-*GFP* in *pSTK*::*bzr1-1D*-*GFP* lines (Figures 3F,G,K,L).

Regarding the study objective, *pSTK*::*bzr1-1D*-*GUS* lines were suitable for the efficient investigation of the BR-specific regulation of reproductive development, but the seed yield of the *pSTK*::*bzr1-1D*-*GFP* lines increased to a greater extent (Figure 3M). The enhanced BR signal in reproductive organs and decreased BR signal in vegetative organs increased the seed yield of individual plants and enhanced the planting density, which considerably increased the total yield of *pSTK*::*bzr1-1D* lines, especially the *pSTK*::*bzr1-1D*-*GFP* lines.

**FIGURE 4:**
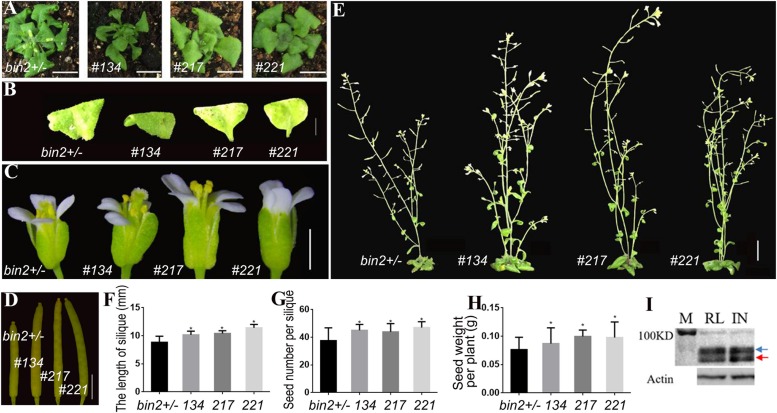
Phenotypic analysis of *pSTK*::*bzr1-1D*-*GFP* lines in *bin2-1* background. **(A,B)** Shape and size of rosette leaves, bar = 1 cm. **(C)** Floral organs, bar = 2 mm. **(D)** Siliques, bar = 2 mm. **(E)** Adult plants, bar = 1 cm. **(F,G)** Silique length and seed number. Bars represent the means ± SD of three biological replicates (*n* = 15). **(H)** Seed weight per plant. Bars represent the means ± SD of three biological replicates (*n* = 5). **(I)** Western-blot showing BZR1 (red arrow) and pBZR1 (blue arrow) in inflorescences (IN) and rosette leaves (RL). The protein marker (M) bands are 100 kD. Actin was the loading control. Asterisks indicate a significant difference (Student’s *t*-test, ^*^P < 0.05).

To further verify that the transgenic lines could differentially regulate the BR signal in various organs and enhance the seed yield, we introduced the *pSTK*::*bzr1-1D*-*GFP* construct into the *bin2-1* mutant. The shape and size of the rosette leaves of three independent transgenic lines (134, 217, and 221) did not change significantly (Figures 4A,B). However, these plants produced larger flowers and siliques (Figures 4C,D,F), grew taller (Figure 4E), and produced more seeds (Figures 4G,H) than the *bin2-1* mutant. A western blot analysis revealed that BZR1 dephosphorylation and the BR signal was greater in reproductive organs than in vegetative organs (Figure 4I), implying that *pSTK*::*bzr1-1D* enhanced the BR signal and seed yield without changing the vegetative organ size of the *bin2-1* mutant.

### Specific Depression of the BR Signal in Reproductive Organs

**FIGURE 5:**
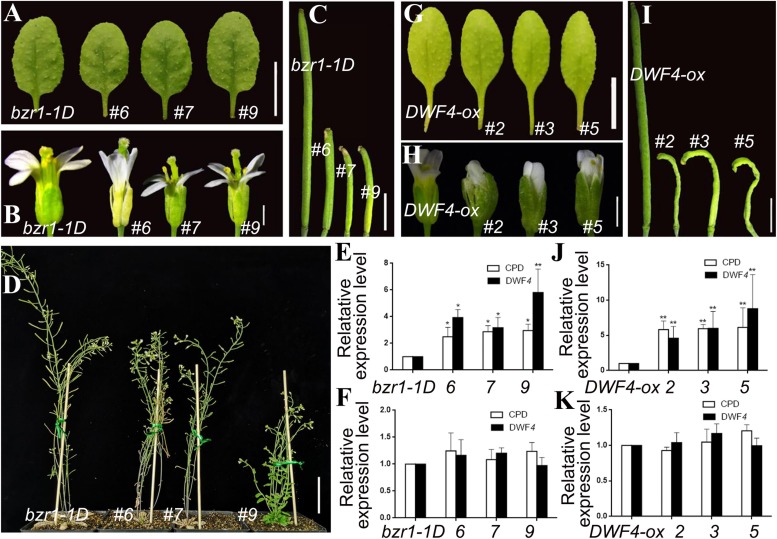
Phenotypic analysis of *pSTK*::*bin2-1-GUS* lines in *bzr1-1D* **(A–F)** and *DWF4-OX* **(G–K)** background. **(A,G)** Shape and size of rosette leaves, bar = 1 cm. **(B,H)** Floral organs, bar = 2 mm **(C,I)** Siliques, bar = 2 mm. **(D)** Adult plants, bar = 5 cm. **(E,J,F,K)** Relative expression level of *CPD* and *DWF4* in inflorescence apex and rosette leaves. Values correspond to the arithmetic means ± SD of three biological replicates (*n* = 3). Asterisks represent significant differences (Student’s *t*-test, ^*^P < 0.05, ^∗∗^*P* < 0.01).

Using a similar method, we incorporated the *pSTK*::*bin2-1*-*GUS* vector into the *bzr1-1D* mutant and *DWF4-OX* plants. Lines 6, 7, and 9 were transgenic lines in the *bzr1-1D* background (Figures 5A–F), whereas lines 2, 3, and 5 were transgenic lines in the *DWF4-OX* background (Figures 5G–K). The transgenic lines produced similar rosette leaves (Figures 5A,G), but smaller floral organs and shorter inflorescence stems (Figures 5B,H), than the control plants. Additionally, the siliques of the transgenic lines were very short and sterile (Figures 5C,I). The height of transgenic lines decreased compared with control plant (Figure 5D). The qRT-PCR results indicated that *CPD* and *DWF4* expression levels in the inflorescence apex were higher in transgenic lines than in the *bzr1-1D* mutant and *DWF4-OX* plants (Figures 5E,J), suggesting that the BR signal was relatively weak in reproductive organs. The transcription of *CPD* and *DWF4* in the rosette leaves was almost unchanged (Figures 5F,K), which indicated that the BR signal was unaffected in vegetative organs. These results implied that we decreased the BR signal specifically in reproductive organs.

**FIGURE 6:**
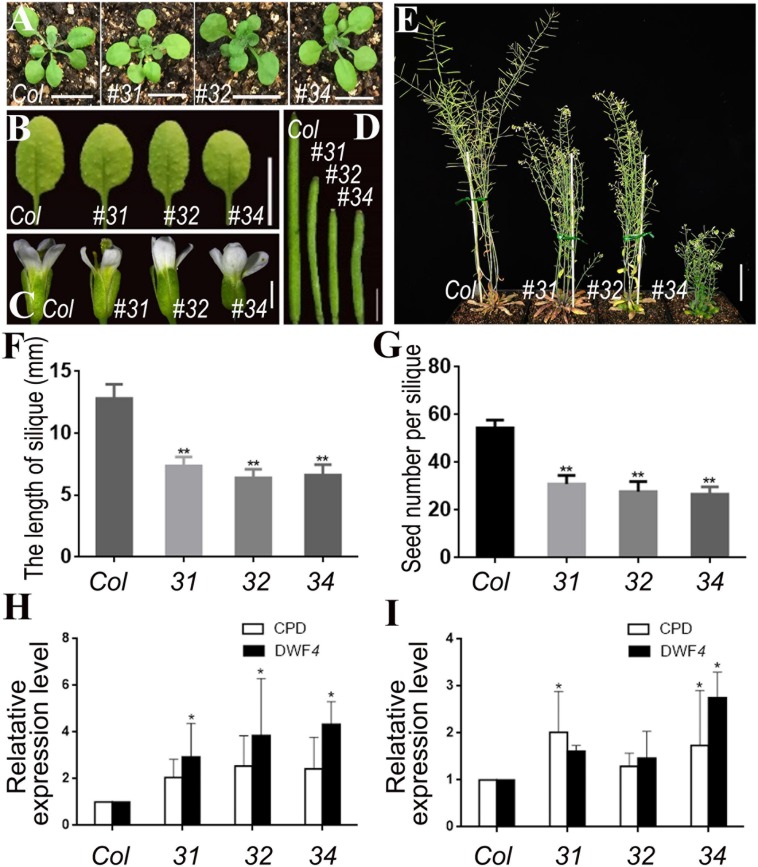
Phenotypic analysis of *pSTK*::*bin2-1*-*GFP* lines in Col background. **(A,B)** Shape and size of rosette leaves, bar = 1 cm. **(C)** Floral organs, bar = 2 mm. **(D)** Siliques, bar = 2 mm. **(E)** Adult plants, bar = 2 cm. **(F,G)** Silique length and seed number. Bars represent the means ± SD of three biological replicates (*n* = 15). **(H,I)** Relative expression level of *CPD* and *DWF4* in inflorescence apex and rosette leaves. Values correspond to the arithmetic means ± SD of three biological replicates (*n* = 3). Asterisks represent significant differences (Student’s *t*-test, ^*^P < 0.05, ^∗∗^*P* < 0.01).

Considering the effect of *pSTK*::*bin2-1*-*GUS* was excessive and led to sterility, we transformed the *pSTK*::*bin2-1*-*GFP* vector into the *bzr1-1D* mutant and *DWF4-OX* plants. However, no significant phenotypic changes were observed in the transgenic lines (Supplementary Figure S4). We then introduced the *pSTK*::*bin2-1*-*GFP* vector into wild-type Arabidopsis (Col) plants. Transgenic lines 31, 32, and 34 exhibited inhibited reproductive development (Figure 6). The shape and size of the rosette leaves did not change significantly (Figures 6A,B), whereas the flower size, silique length, plant height, and number of seeds decreased (Figures 6C–G) relative to the corresponding values for the wild-type control. The qRT-PCR results revealed that the *CPD* and *DWF4* transcript levels in the inflorescence apex were higher in the transgenic lines than that in Col (Figure 6H). The data also suggested that the BR signal was weaker in reproductive organs than in vegetative organs. The *CPD* and *DWF4* transcript levels in rosette leaves were higher in the transgenic lines than in the control plants (Figure 6I), indicating that the BR signal also changed in the rosette leaves, but not as markedly as in the inflorescence apex. These results also confirmed that we specifically decreased the BR signal in reproductive organs.

## Discussion

The regulation of BR is difficult to manipulate because of the complexity of the BR signal. A previous study (Wang et al., 2008) suggested that BR can enhance rice yield by upregulating the expression of BR biosynthesis genes driven by an artificial promoter with an enhancer. The promoter used in this earlier study was not a reproductive tissue-specific promoter, but was active in vegetative organs. The authors proposed that the BR signal stimulated the flow of assimilates from the source to the sink, ultimately resulting in enhanced rice yield. Moreover, they predicted that the expression of the same gene driven by an embryo-specific promoter in transgenic lines would not affect rice yield. The modulation of BR biosynthesis gene expression with a tissue-specific promoter does not appear to alter the BR signal in specific organs. In our study, the coding sequences directly regulated by the BR signal (*bzr1-1D* and *bin2-1*) and driven by tissue-specific promoters enhanced the BR signal only in the reproductive organs of transgenic lines, indicating the BR signal could be accurately manipulated in specific organs. The ideal combination (Figure 1A, R1V2) for increasing seed yield was determined, and the transgenic systems functioned as predicted.

The *STK* promoter is predicted to be highly active in various organs, including vegetative and reproductive organs, but especially in the pistil and ovule (Supplementary Figure S1). However, previously reported GUS assay results (Kooiker et al., 2005) and our results suggest that *STK* transcription is limited to the septum and ovule. The expression of our coding sequences driven by the *STK* promoter enhanced the BR signal in reproductive organs. Additionally, the two vectors used in this study differed regarding efficiency. For example, the enhanced BR signal was limited to flowers and siliques in the *pSTK*::*bzr1-1D*-*GUS* lines, and the adult plants were slightly taller. In contrast, the enhanced BR signal spread to additional organs in the *pSTK*::*bzr1-1D*-*GFP* lines, and the adult plants were considerably taller, with the architecture of some lines resembling that of wild-type plants. It seemed that the *STK* promoter was highly and more widely active in GFP vector than GUS vector, which was consistent with the predicted transcription based on electric northern (Supplementary Figure S4). The *pSTK*::*bzr1-1D*-*GFP* lines exhibited a greater expansion of the BR signal (i.e., in reproductive and vegetative organs) for unreported reasons. Fortunately, the BR signal was preferentially enhanced in the reproductive organs of *pSTK*::*bzr1-1D*-*GFP* lines, which indicated that the transformation system functioned as intended. Additionally, the seed yield of *pSTK*::*bzr1-1D*-*GFP* lines was considerably higher than that of *pSTK*::*bzr1-1D*-*GUS* lines.

Moreover, the efficiency of the *pSTK*::*bin2-1* vectors varied. Inflorescence growth was suppressed in the transgenic lines of the *bzr1-1D* mutant and *DWF4-OX* plants harboring pBI101.3 vectors. But the transgenic lines are sterile. In the opposite way, there were no significant phenotypic changes in the transgenic lines harboring *pSTK*::*bin2-1*-*GFP* in *DWF4-OX* and *bzr1-1D* backgrounds. The BR signal may have been too strong to be suppressed in the *DWF4-OX* and *bzr1-1D* backgrounds. However, *pSTK*::*bin2-1*-*GFP* was effective in the Col background, suggesting that it could be used to study the BR-specific regulation of reproductive development. Theoretically, this system may be exploited to shorten inflorescences and enhance lodging resistance via appropriate modifications.

Fortunately, the *pSTK*::*bin2-1*-*GFP* lines in the Col background suppressed the reproductive development and produced less seed. The pBI101.3 vector was associated with specific *STK* promoter activity, resulting in the highly efficient modulation of the BR signal in the expected organs. In contrast, the pCAMBIA1302 vector was associated with expanded *STK* promoter activity and the relatively less-specific regulation of the BR signal. Considering this system may be used for future investigations and crop improvement, the combinations appropriate for use will depend on the specific aims.

In this study, we used a reproductive tissue-specific promoter (*pSTK*), coding sequences for modulating the BR signal (*bzr1-1D* and *bin2-1*), and vectors differing in efficiency (pBI101.3 and pCAMBIA1302) to preferentially modulate the BR signal in reproductive organs. The transgenic constructs enabled the differential regulation of the BR signal in vegetative and reproductive organs, which could be useful for studying the BR-specific regulation of reproductive development and for improving crop production. We increased flower/fruit size, seed production, and yield without affecting the vegetative organ size and planting density. On the basis of our results, the ideal procedure involves the introduction of *pSTK*::*bzr1-1D*-*GFP* into the weak allele of BR-deficient/insensitive plants to increase reproductive organ size and decrease vegetative organ size, and consequently enhance total yield by increasing the seed yield of individual plants and increasing planting density (Figure 1A, R1V2). Additional combinations of promoters, coding sequences, vectors, and genotypic backgrounds can be optimized in the future to study the BR-specific regulation of various developmental stages and potentially improve crop yield.

## Data Availability

All datasets generated for this study are included in the manuscript and/or the Supplementary Files.

## Author Contributions

W-HL designed the study, wrote and modified the manuscript, and acquired funding. S-HZ performed the experiments, organized the figures, and wrote the manuscript. Y-TJ, L-QH, and J-HC completed the experiments. Y-JZ helped to modify the figures. H-WX helped to organize the results. All authors agreed to be accountable for the contents of this manuscript.

## Conflict of Interest Statement

The authors declare that the research was conducted in the absence of any commercial or financial relationships that could be construed as a potential conflict of interest.
