# Supplementary figures and images for: Effective Modulating Brassinosteroids Signal to Study Their Specific Regulation of Reproductive Development and Enhance Yield
Source: Front Plant Sci. 2019 Jul 26;10:980. doi: 10.3389/fpls.2019.00980 (PMC6676975; doi:10.3389/fpls.2019.00980)

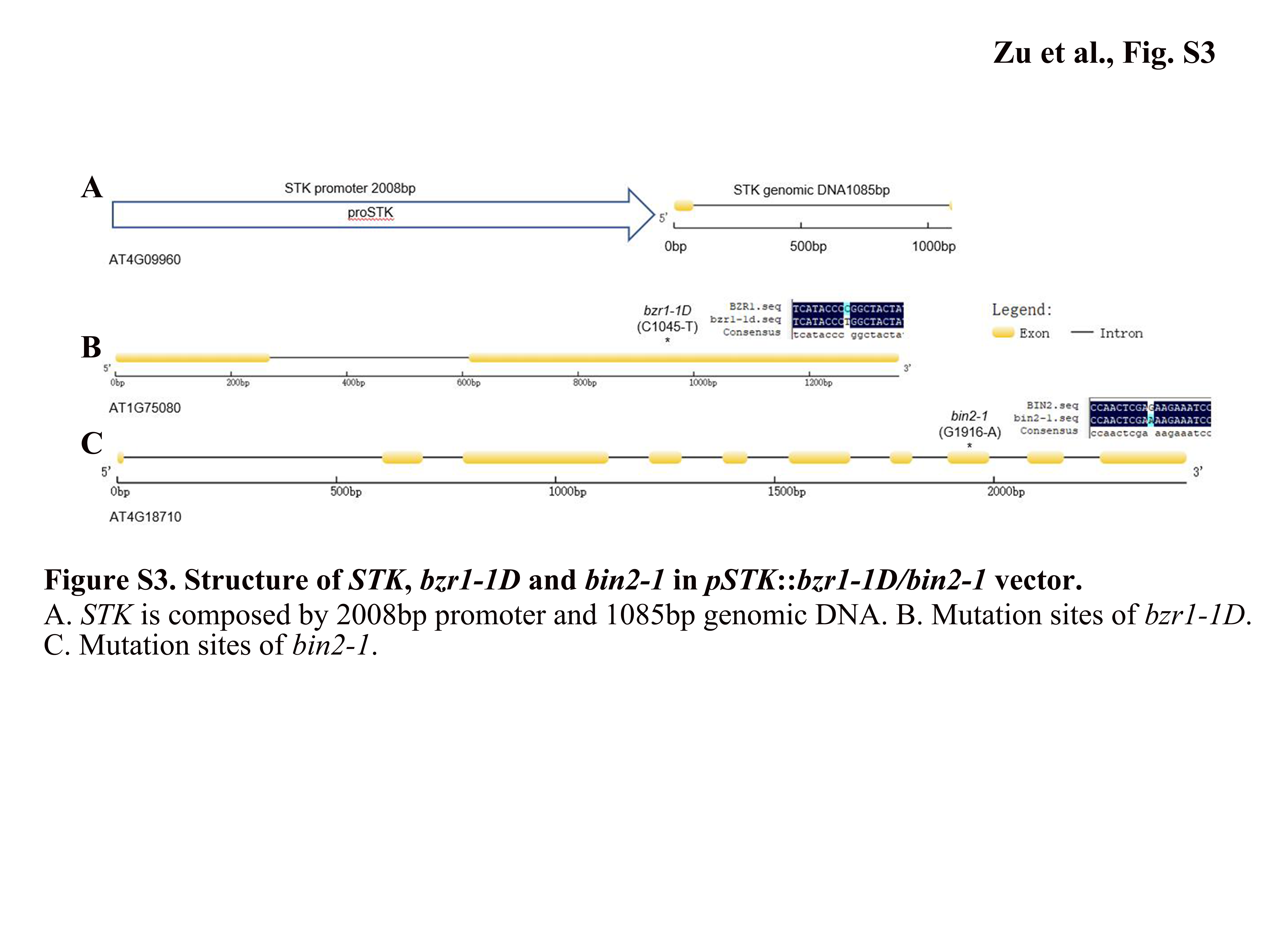

Supplement: Supplementary file 3 [file Image_3.TIF]
