# Supplementary material for: Effective Modulating Brassinosteroids Signal to Study Their Specific Regulation of Reproductive Development and Enhance Yield
Source: Front Plant Sci. 2019 Jul 26;10:980. doi: 10.3389/fpls.2019.00980 (PMC6676975; doi:10.3389/fpls.2019.00980)
Supplement: Supplementary file 5 [file Table_1.pdf]

Supplementary Table S1. Primer sequences for construct PBI101.3 STK-GUS, PBI101.3 STK-*bzr1-1D*-GUS, PCAMBIA1302 STK-*bzr1-1D*-GFP, PBI101.3 STK-*BIN2-1*-GUS and PCAMBIA1302 STK-*BIN2-1*-GFP.

Name	Sequence 5'-3'
STK-F	CGACTCTAGAGGATCCCCGCTCTGCAATTTCACCTTTCTCTT
STK-F1	TATGACCATGATTACGAATTCGCTCTGCAATTTCACCTTTC
STK-R	CCGTACCCGGGGATCCTCCTTCATTTTAAACATCAAAC
STK-R1	GTAGCTCCATCCGAAGTCATTCTAGACCTTCATTTTAAACATCAAAC
STK-R2	CAGCTTTACCTTATCATCAGCCATTCTAGACCTTCATTTTAAACATCAAAC
BZR1-F	ATGACTTCGGATGGAGCTACGT
BZR1-R	ACCACGAGCCTTCCCATTTCCA
bzr1-1D-F	CAGTTTCATACCCTGGCTACTATA
bzr1-1D-R	TATAGTAGCCAGGGTATGAAACTG
bzr1-1D-F1	GTTTAAAATGAAGGTCTAGAATGACTTCGGATGGAGCTACGT
bzr1-1D-R1	CCGTACCCGGGGATCCTACCACGAGCCTTCCCATTTCCA
bzr1-1D-R2	AGATCTACCATGGCTCTAGAACCACGAGCCTTCCCATTTCCA
BIN2-F	ATGGCTGATGATAAGGTAAAGCTG
BIN2-R	AGTTCCAGATTGATTCAAGAAGCT
BIN2-1-F	ACCAACTCGAAAAGAAATCCGTT
Bin2-1-R	AACGGATTTCTTTTCGAGTTGGT
BIN2-1-F1	GTTTAAAATGAAGGTCTAGAATGGCTGATGATAAGGTAAAGCTG
BIN2-1-R1	CCGTACCCGGGGATCCTAGTTCCAGATTGATTCAAGAAGCT
BIN2-1-R2	AGATCTACCATGGCTCTAGAAGTTCCAGATTGATTCAAGAAGCT

Supplementary Table S2. Primer sequences for qRT-PCR assay

Name	Sequence 5'-3'
CPD RT-F	CATGGAAGAAGCCAAAAAGATAACG
CPD RT-R	CTTTGCGGTAAGTGGTGGAGA
DWF4 RT-F	CATGTCTCCAAGTATGGTAAGATAT
DWF4 RT-R	ATTTCCCAAGAATCCCACCTATACT
bzr1-1D RT-F	TACTGCTGCCTTCCAAGAGATT
GFP RT-R	CATCACCTTCACCCTCTCCACT
ACTIN RT-F	CCGGTATTGTGCTCGATTCTG
ACTIN RT-R	TTCCCGTTCTGCGGTAGTGG

Supplementary Table S3. Primer sequences for identification transgenic lines

Name	Sequence 5'-3'
77-R	TGTCCTTGAGATCAATCAACA
GUS	AGACTTCGCGCTGATACCAG
GFP	GAACACCATAAGAGAAAGTAGTG
